# Microanatomical study of arachnoid granulations and meningeal architecture around Meckel’s cave

**DOI:** 10.1007/s10143-023-01954-0

**Published:** 2023-02-09

**Authors:** Grzegorz Wysiadecki, R. Shane Tubbs, Joe Iwanaga, Maciej Radek, Jerzy Walocha, Piotr Brzeziński, Józef Kobos, Michał Polguj

**Affiliations:** 1 Department of Normal and Clinical Anatomy, Chair of Anatomy and Histology, Medical University of Lodz, Ul. Żeligowskiego 7/9, 90–752 Lodz, Poland; 2 Department of Neurosurgery, Tulane Center for Clinical Neurosciences, Tulane University School of Medicine, New Orleans, LA USA; 3 Department of Neurology, Tulane Center for Clinical Neurosciences, Tulane University School of Medicine, New Orleans, LA USA; 4 Department of Anatomical Sciences, St. George’s University, St. George’s, Grenada; 5 Department of Structural & Cellular Biology, Tulane University School of Medicine, New Orleans, LA USA; 6 Department of Surgery, Tulane University School of Medicine, New Orleans, LA USA; 7 Department of Neurosurgery and Ochsner Neuroscience Institute, Ochsner Health System, New Orleans, LA USA; 8 University of Queensland, Brisbane, Australia; 9 Department of Neurosurgery, Spine and Peripheral Nerve Surgery, Medical University of Lodz, University Hospital WAM-CSW, Lodz, Poland; 10 Department of Anatomy, Jagiellonian University Medical College, Cracow, Poland; 11 Department of Histology and Embryology, Chair of Anatomy and Histology, Medical University of Lodz, Lodz, Poland

**Keywords:** Arachnoid granulations, Meckel’s (trigeminal) cave, Meninges, Microsurgical anatomy, Skull base, Trigeminal nerve, Trigeminal (Gasserian) ganglion

## Abstract

Although the microanatomy of Meckel’s cave (MC) has been well studied, there are still controversies regarding the meningeal architecture of the space. Moreover, there are only general mentions of the arachnoid granulations near MC in just a few sources. This study is aimed at determining the frequency, location, and anatomical variability of the main clusters of arachnoid granulations around MC. The dissection involved 26 isolated specimens of MC fixed in formalin (neutral buffered, 10%). This number included five freshly harvested specimens examined histologically. Additional paraffin block with MC horizontal section was taken from our neuroanatomical collection. Carefully selected anatomical and histological techniques were applied to assess the complex relationships between the arachnoid granulations and adjacent structures. Arachnoid granulations were found around MC in all specimens with different anatomical variations. The main clusters of arachnoid granulations were close to the trigeminal ganglion and its divisions. The dorsolateral wall of MC was a thick layer formed by interweaving bundles of collagen fibers arranged in various directions. The entire MC was surrounded by a dural sleeve (envelope). This sleeve separated MC from the lateral sellar compartment. At its anterior (rostral) end, it formed a cribriform area pierced by individual fascicles of the trigeminal nerve’s primary divisions. The connective tissue forming the sleeve was not only continuous with the epineurium but also shifted to the perineuria surrounding individual nerve fascicles. The meningeal architecture around MC has a complex and multilayer arrangement with a collagenous sleeve closely related to the trigeminal ganglion. Arachnoid granulations are typically found around MC.

## Introduction

Meckel’s cave (MC), also known as the trigeminal cave, is a cerebrospinal fluid-containing dural recess that occupies the posterolateral aspect of the parasellar compartment. The trigeminal nerve enters the cave from the prepontine cistern. The cave contains the trigeminal triangular plexus [2] and trigeminal (Gasserian or semilunar) ganglion, where the trigeminal nerve’s primary divisions originate. The space is adjacent to the lateral and posterior walls of the cavernous sinus and is one of the areas often affected by tumor metastasis, which makes it relevant for surgical planning and diagnostic imaging [1, 27]. As Cheung et al. [6] stressed, “Meckel’s cave is an avenue for tumors to spread between the posterior and middle cranial fossae,” trigeminal schwannomas and meningiomas being the most common neoplasms transversing this channel.

Although the microanatomy of MC has been well studied, there are still controversies regarding the meningeal architecture of the space [3, 32]. Moreover, there are brief mentions of the arachnoid granulations (also known as the Pacchionian granulations) in the proximity of MC in only a few sources [3, 16, 19, 20, 32]. The arachnoid granulations could suggest a role for MC as a cerebrospinal fluid absorbent [19], which could be of considerable physiological and clinical significance. This study is aimed at determining the frequency, location, and anatomical variability of the main clusters of arachnoid granulations around MC. Carefully selected anatomical and histological techniques were applied to assess the complex relationships between the arachnoid granulations and adjacent structures. Of particular importance was whether arachnoid granulations around MC are normal or aberrant anatomical features. Immunohistochemical techniques were applied to trace the relationships between the granulations and venous channels around MC. Finally, the distribution of meningeal walls was observed using specialized histochemical techniques. The authors hope that this work will constitute another piece in an anatomical puzzle that will help elucidate the microanatomy of MC. The article is supplemented with extensive illustrations and a discussion of the possible significance of the findings.

## Material and methods

### Microanatomical dissection

**Fig. 1:**
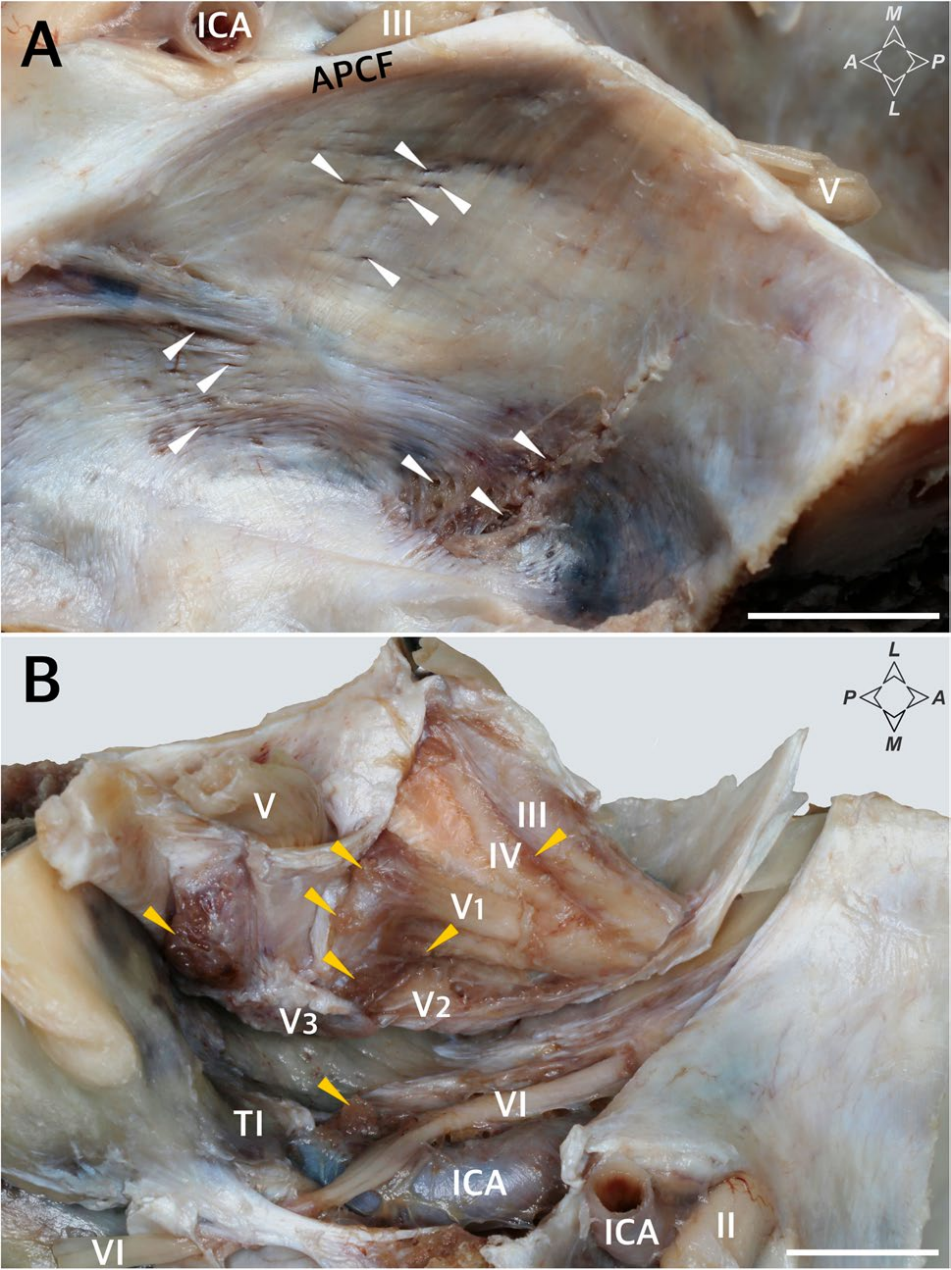
Dissection and topographical anatomy of the left Meckel’s cave. **A** Superolateral view of the dorsolateral wall of MC with numerous tiny pores and trabeculations (marked by white arrowheads). **B** Superomedial view of the inferomedial wall of MC. Meckel’s cave is separated from the trigeminal impression (TI). The content of the lateral sellar compartment is exposed. Few clusters of arachnoid granulations (marked by yellow arrowheads) are located along the rostral end of MC, at the origins of the ophthalmic (V1) and maxillary (V2) nerves, and between the two divisions. Arachnoid granulations are also present around the trochlear (IV) and oculomotor (III) nerves and posterior to the mandibular nerve (V3). II, optic nerve; V, trigeminal nerve; VI, abducens nerve; APCF, anterior petroclinoid fold (ligament); ICA, internal carotid artery. Directions: A, anterior; P, posterior; L, lateral; M, medial. The scale bar shows 10 mm

The study was conducted on 21 specimens of MC obtained from sagittal sections of the head (nine specimens harvested from the right side and 12 from the left) fixed in formalin (neutral buffered, 10%). The ages and medical histories of the donated bodies were unknown since the head specimens were isolated. However, signs of head trauma or neurosurgical interventions were excluded. Each sample of MC was taken from a different body donor. Microsurgical equipment was used for dissection, under magnification with a binocular loupe. A dural incision was made along the interclinoid dural fold, involving the posterior petroclinoid dural fold, which was cut and was then extended along (a few millimeters below) the posterior petroclinoid dural fold inferior to the porus trigeminus. The periosteal dura was gently separated using a double-ended Molt periosteal elevator, which exposed the inferomedial aspect of Meckel’s cave (the dural cuff covering the porus trigeminus was preserved) and the parasellar space (Fig. 1). The presence and distribution of the main clusters of arachnoid villi were assessed topographically in relation to the trigeminal ganglion and its divisions. The surroundings of MC (especially the opening of the superior petrosal sinus) were also checked for arachnoid granulations.

### Histological examination

Another five freshly harvested specimens (two right and three left) obtained up to 72 h after death underwent gross anatomical and histological examination. The donors’ age for this sample ranged between 47 and 84 years (mean, 67 years; SD = 13). After fixation in 10% neutral buffered formalin (equivalent to 4% paraformaldehyde), the distribution of arachnoid granulations was assessed. The specimens were then embedded in paraffin and sliced with a rotatory microtome into 5-µm-thick slices for histochemical staining or 3-µm-thick slices for immunohistochemistry. Two MC samples were embedded and sectioned in the sagittal plane and another three in the horizontal plane. Histological specimens perpendicular to the porus trigeminus and the maxillary and mandibular nerves were also prepared from the material intended for horizontal sections. An additional paraffin block with MC horizontal section was taken from our neuroanatomical collection. A total of six different MC specimens were evaluated histologically. Various histological techniques were applied to assess the microanatomical relationships. H&E staining was routinely used for all specimens. The Masson–Goldner trichrome was used on selected samples to visualize the connective tissue. Since the specimens were fixed in formalin, samples selected for the Masson–Goldner trichrome staining were incubated in Bouin’s solution (1 h at 60 °C). Picrosirius red stain (also called “Sirius red” stain) helped trace the distribution of collagenous structures (e.g., meningeal coverings of MC and the epineuria and perineuria of nerves). Collagen fibers were detected in tissue sections by picrosirius red polarization following the protocol described by Rittié [31]. Automated immunohistochemistry was used to highlight the vascular endothelium and check the relationships between arachnoid granulations and venous channels. Anti-CD31 immunostaining (monoclonal mouse anti-human CD31 antibody, Clone JC70A, Dako) was applied to detect vascular endothelium. Additionally, anti-D2-40 immunostaining (monoclonal mouse anti-human podoplanin antibody, Clone D2-40, Dako) was used to mark the lymphatic endothelial cells. A Dako Autostainer Link 48 was used for automated immunohistochemistry, following the previously described protocol [10].

### Image acquisition

Histological specimens were assessed and photographed using an OPTA-TECH MB 200 series biological microscope (OPTA-TECH, Warsaw, Poland) with an OPTA-TECH HDMI CAM microscope camera with HDMI CAM-embedded software. Picrosirius red-stained sections were examined and photographed under an Olympus BX50 fluorescence microscope with an installed camera (DP71), Olympus U-POT polarizer, and Olympus U-ANT analyzer.

## Results

### Gross anatomical observations on the distribution of arachnoid granulations

**Fig. 2:**
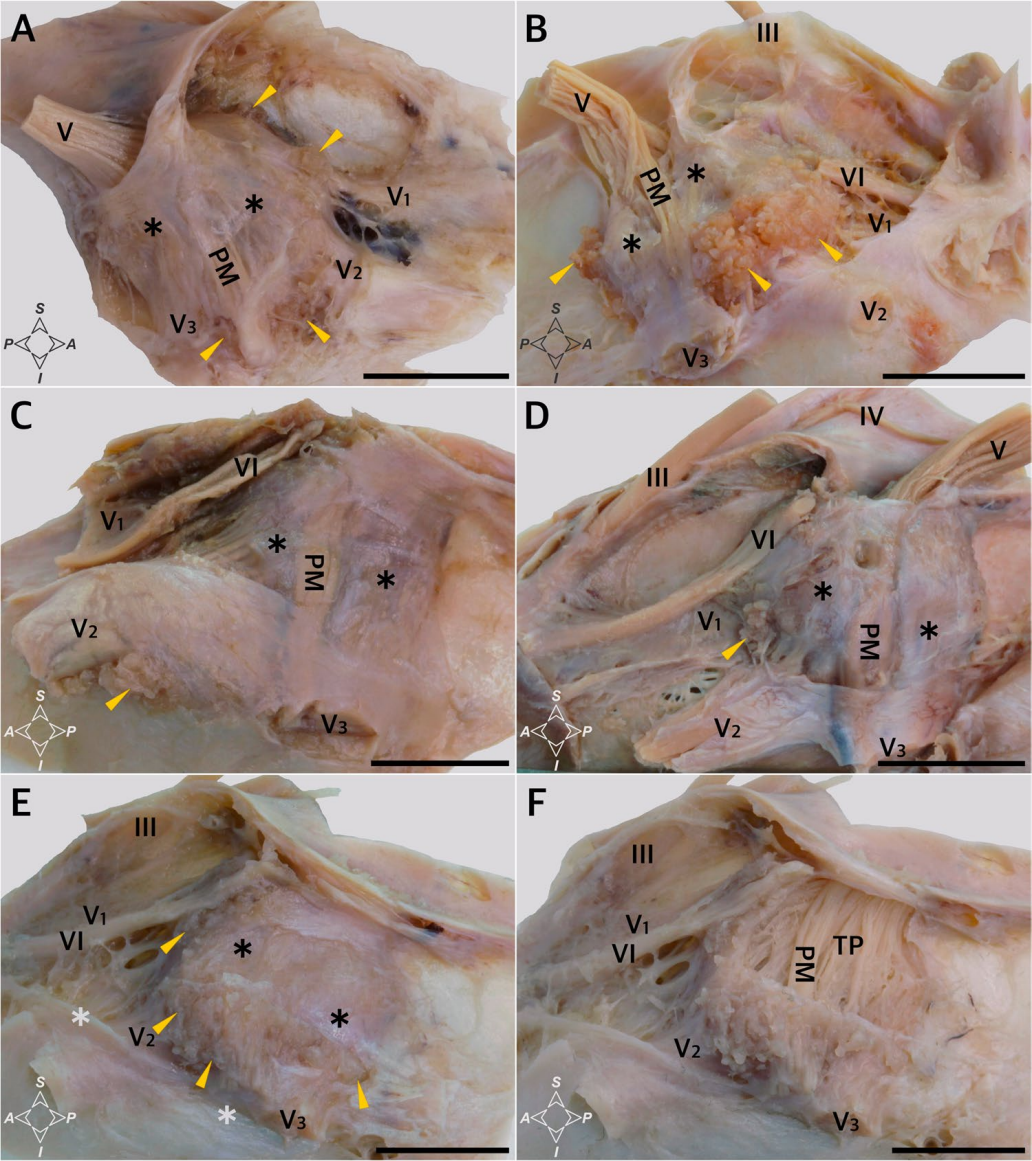
Topographical anatomy, meningeal architecture, and various patterns of arachnoid granulations distribution around MC. Black asterisks mark the dural sleeve of Meckel’s cave. In the specimen shown in **A**, arachnoid granulations (marked by yellow arrowheads) are distributed around the origin of the ophthalmic division (V1) and between the maxillary (V2) and mandibular (V3) divisions of the trigeminal nerve (V). **B** Well-developed clusters of arachnoid granulations are located around the V1 division, between V1 and V2, between V2 and V3, and posterior to V3. **C** A large cluster of arachnoid granulations is located exclusively between the V2 and V3 divisions. In this variant, surgical access through the anterolateral triangle can be hindered. **D** A small cluster of arachnoid granulations is located exclusively between the V1 and V2 divisions. **E** and **F** show the same specimen. In **E**, the dural sleeve of MC was preserved. Arachnoid granulations are distributed along the trigeminal ganglion out of the dural sleeve. Prominent clusters of granulations are visible around the V1 division (but not between V1 and V2); V2, between V2 and V3; and posterior to V3. VI, abducens nerve; PM, portio minor (motor part) of the trigeminal nerve. White asterisks mark the periosteal dura incised during specimen harvesting. In **F**, the dural sleeve of MC was removed, and the trigeminal triangular plexus (TP) was exposed. **A** and **B** show specimens harvested from the left side, while **C–F** show specimens harvested from the right. Directions: A, anterior; P, posterior; S, superior; I, inferior. The scale bar shows 10 mm

**Fig. 3:**
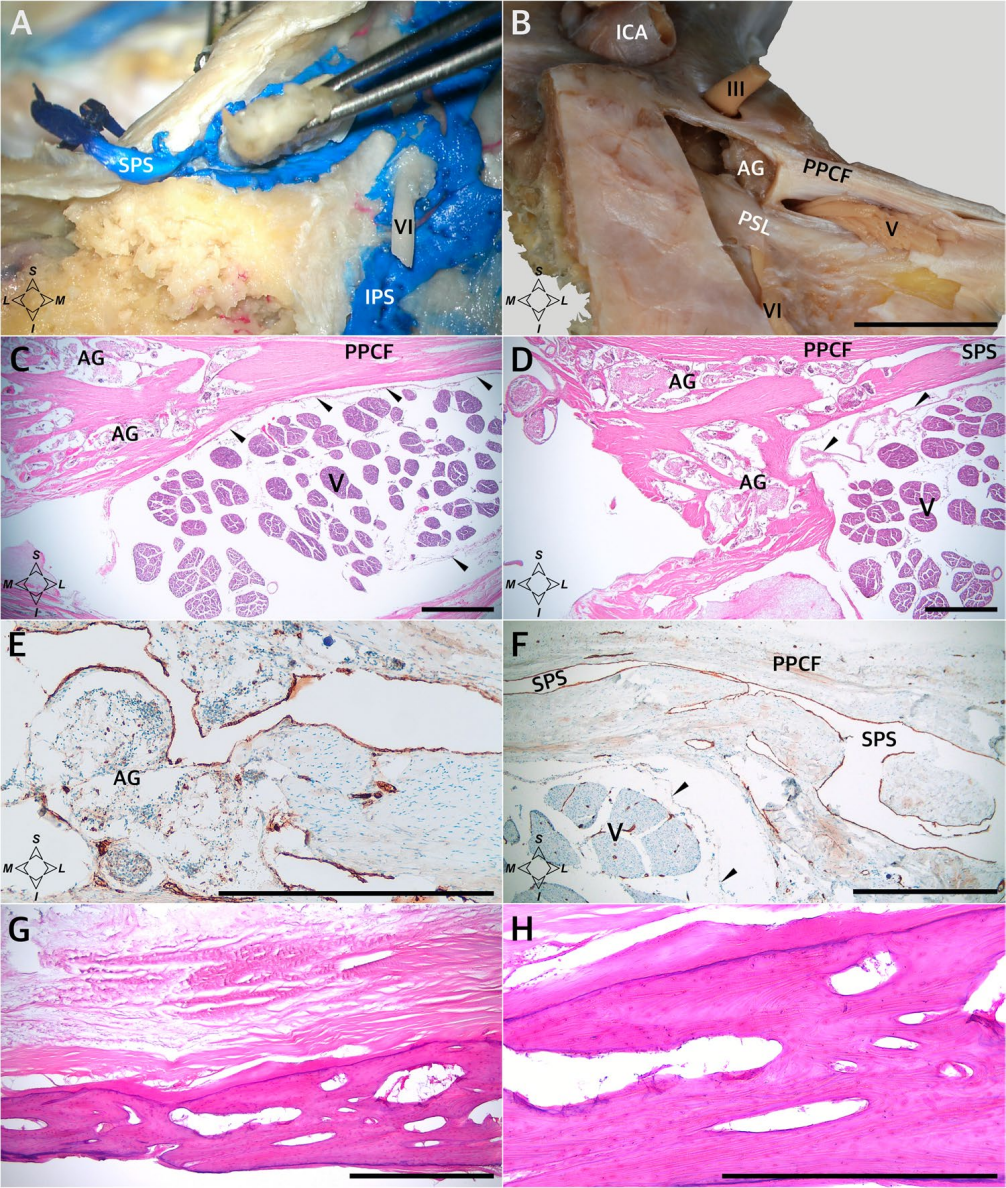
Topographical relationships around the porus trigeminus. **A** Silicone-injected specimen showing superior petrosal sinus (SPS) around the porus trigeminus. Posterior view of the left side. The trigeminal nerve is held with tweezers. **B** Posterior view of the right side of another specimen. The inferomedial paraclival triangle was exposed by removing the corresponding dura. A large cluster of arachnoid granulations (AG) is revealed in the upper lateral corner of the triangle, on the medial side of the porus trigeminus. In **B**, the scale bar shows 10 mm. **C** Histological specimen of the dural entry point to the right of Meckel’s cave. H&E stain. Aggregation of AG is shown on the medial side of the porus trigeminus in the SPS opening. The posterior petroclinoid dural fold (PPCF) forms the roof of the porus. Arachnoid mater is marked by black arrowheads. **D** Microsopic view of deeper layer of the same sample. AG are visualized in the SPS opening. **E** The same sample. Anti-CD31 immunostaining revealed vascular epithelium (stained brown by DAB) surrounding venous channels (VC) or lacunae around AG. **F** Anti-CD31-immunostained specimen showing the lateral side of the porus trigeminus and its relationship to the SPS, which is lined with vascular epithelium (dark brown). Numerous vascular vessels are visible in the trigeminal nerve (V) stem. **G** Heterotopic bony tissue among collagen fiber bundles forming the posterior petroclinoid dural fold. Longitudinal section of the fold. Sample taken over the porus trigeminus. H&E stain. **H** Magnification of **G** showing compact bone structure. In **C–H**, the scale bar shows 1 mm. ICA, internal carotid artery; PSL, petrosphenoidal (petroclival) ligament; III, oculomotor nerve; VI, abducens nerve. Directions: L, lateral; M, medial; S, superior; I, inferior

Arachnoid granulations were found around MC in all specimens examined (Figs. 1 and 2). However, the distribution of these granulations was not homogeneous, and different anatomical variations were observed in their arrangement. The distribution of the main clusters of arachnoid granulations in relation to the trigeminal ganglion branches, third and fourth cranial nerves, and opening of the superior petrosal sinus is presented in Table 1 and Fig. 2. The granulations were scattered around the trigeminal ganglion and its branches. In most cases (18/26; 69.2%), well-developed clusters were found in the triangular space between the V2 (maxillary) and V3 (mandibular) divisions of the trigeminal nerve (Table 1; Fig. 2a–c, e, f), representing the anterolateral triangle (the triangle is delineated by the posterior border of the maxillary division, the anterior border of the mandibular division, and a line connecting the foramen rotundum with foramen ovale). Arachnoid granulations in the triangular space between the V1 (ophthalmic) and V2 divisions, representing the anteromedial triangle (delineated by the lower margin of the ophthalmic division and the upper margin of the maxillary division), were less common (11/26 specimens; 42.3%; Table 1; Figs. 1 and 2b, d). In most cases, clusters of granulations did not appear to affect the areas of surgical access significantly within the anteromedial and anterolateral triangles. They formed relatively small conglomerates, not exceeding the upper corners of both triangles. However, in one specimen (1/26; 3.8%), there was a large cluster of granulations between the V2 and V3 divisions, reducing the area of the anterolateral triangle (Fig. 2c). Well-developed aggregations of arachnoid granulations posterior to the maxillary nerve and trigeminal ganglion were found in eight of the 26 sides (30.8%, Figs. 1 and 2b, e). In contrast, there were arachnoid granulations in the opening of the superior petrosal sinus in 13 of the 26 specimens (50%, Fig. 3b), which was also confirmed histologically (Fig. 3c–e). These clusters of granulations were located on the medial side of the porus trigeminus (trigeminal nerve entrance to Meckel’s cave; see Fig. 3). On the basis of topographic criteria, arachnoid granulations in the opening of the superior petrosal sinus could be considered contents of the inferolateral paraclival triangle and could extend as far as the upper lateral corner of the inferomedial paraclival triangle (Fig. 3b–d).

**Table 1:** The distribution of the main arachnoid granulations (AG) clusters in relation to the branches of the trigeminal ganglion (TG), third (CN III) and fourth (CN IV) cranial nerves, and the opening of the superior petrosal sinus (SPS) observed macroscopically

No	Side	Location of standout AG clusters					
		Around V1 origin	Between V1 and V2	Between V2 and V3	Behind V3	CN III and IV	SPS opening
1	L	X	X	X	X	X	X
2	R			X	X		
3	L	X		X			X
4	R	X		X	X		X
5	L	X	X	X	X		
6	R		X				
7	R		X	X			
8	R	X		X			X
9	L	X	X	X		X	X
10	L			X		X	
11	R			X			
12	L	Small AG equally distributed along TG			X		
13	L		X		X		
14	L	Small AG equally distributed along TG					
15	L			X	X		
16	R	Small AG equally distributed along TG				X	X
17	L			X	X		
18	L	X	X			X	X
19	R	X	X				X
20	L	X		X			X
21	R	X		X			X
22	L		X			X	
23	R	X		X			X
24	L	X	X	X			
25	R			X		X	X
26	L	X	X	X		X	X

### Histological observations of the arachnoid granulations

**Fig. 4:**
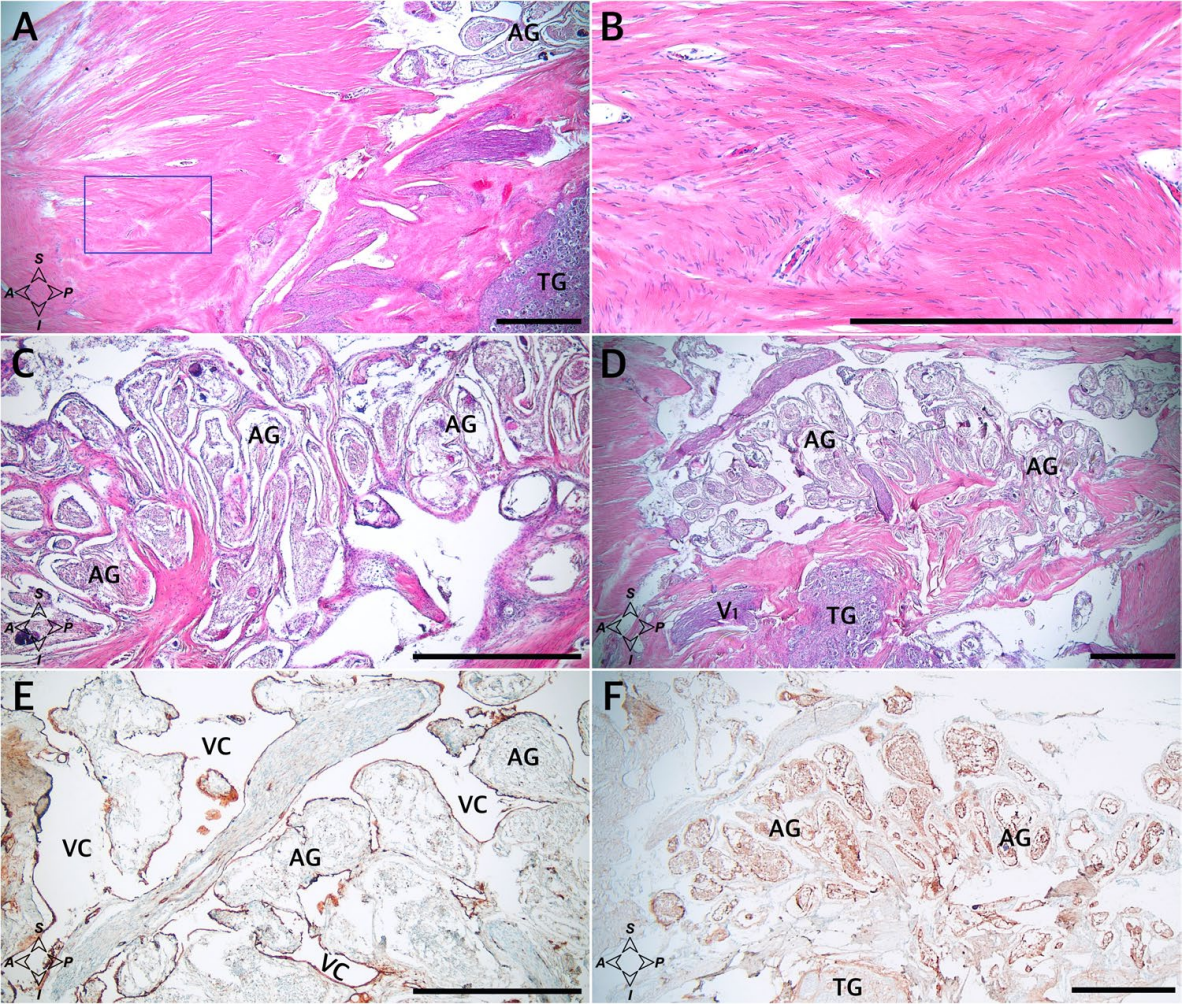
Sagittal sections of the dorsolateral wall of Meckel’s cave. **A** Distributions of collagen fiber bundles. H&E stain. Arachnoid granulations (AG) and the trigeminal ganglion (TG) are partially exposed. **B** Magnification of the area marked with the blue rectangle in **A**. Rows of fibrocytes show the course of interweaving collagen fiber bundles arranged in various directions. **C** A large cluster of AG revealed during further trimming of the dorsolateral (superolateral) wall of MC, just under this wall. H&E stain. **D** A deeper (more medial) layer of the same area. H&E stain. **E** Anti-CD31-immunostained specimen from the same sample. Venous channels (VC) are revealed around the AG. These channels can be considered parts of the laterotrigeminal venous system. The vascular epithelium is stained dark brown. **F** Anti-D2-40-immunostained specimen from the same sample with arachnoid granulations stained positively. Directions: A, anterior; P, posterior; S, superior; I, inferior. The scale bar shows 1 mm

**Fig. 5:**
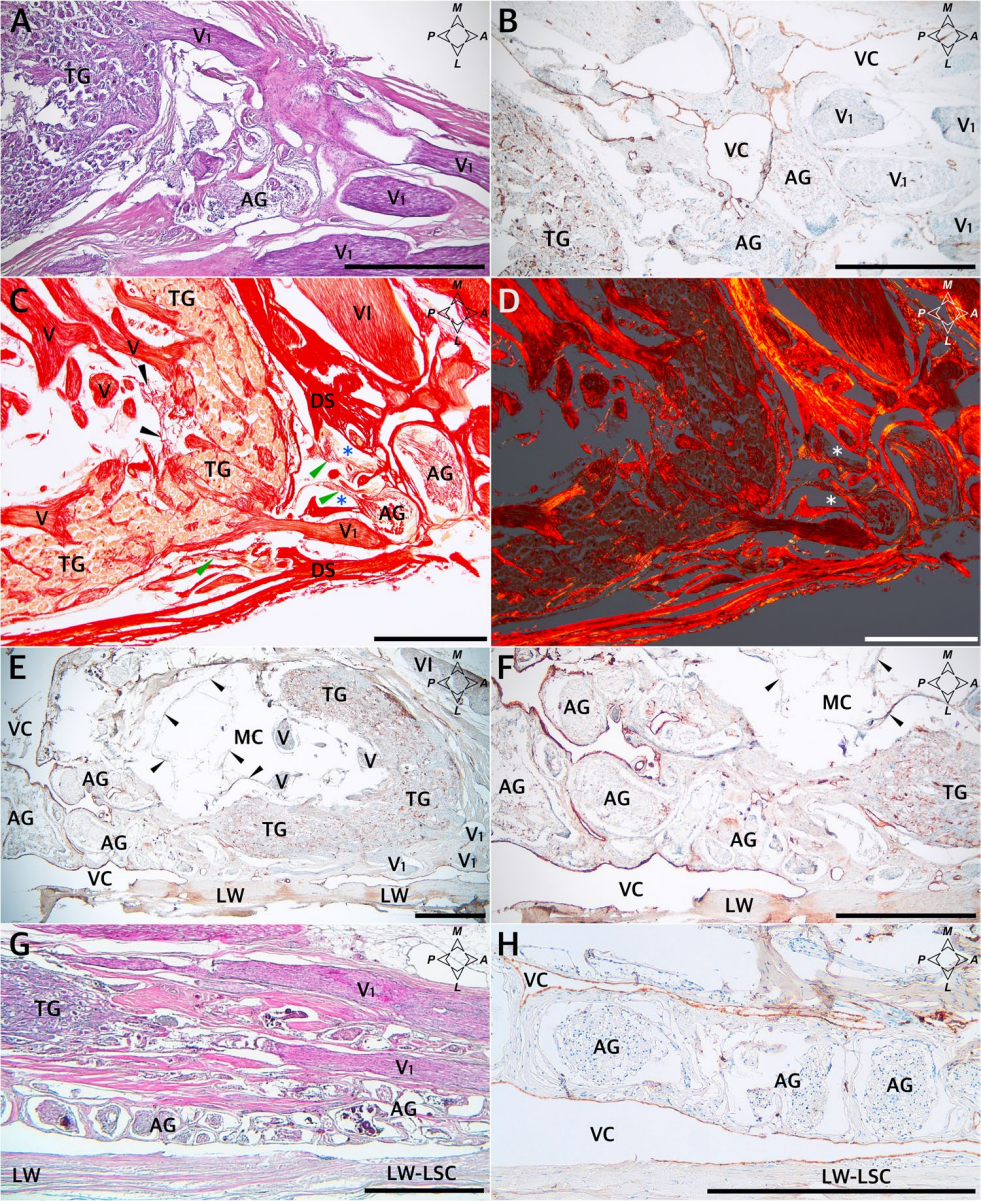
Horizontal sections of three different histological specimens showing arachnoid granulations (AG) around Meckel’s cave. **A–D** Arachnoid granulations near the anterior pole of the trigeminal ganglion and trigeminal nerve’s ophthalmic division (V1). In **A**, AG are located anterior to the distal edge of the trigeminal ganglion (TG) and Meckel’s cave, between individual fascicles of V1. H&E stain. **B** The same sample, anti-CD31 immunostained. AG are surrounded by venous channels (VC). The vascular endothelium is stained brown. **C** The same specimen stained with picrosirius red and seen under normal light. AG near the origin of the ophthalmic nerve (V1) and their relationships to the dural sleeve (DS) of MC are presented. Black arrowheads indicate arachnoid mater on the inner side of the trigeminal ganglion (TG). Blue asterisks indicate a gap in the DS enabling communication to be made with AG—a potential space for cerebrospinal fluid outflow from MC. Green arrowheads mark the arachnoid mater on the external surface of the TG. V, fascicles of the trigeminal nerve located in MC; VI, abducens nerve. **D** The same specimen seen under polarized light. Meningeal relationships are highlighted owing to the natural birefringence of collagen fibers exposed to polarized light. AG are located on the external surface of the DS. The TG adheres closely to the internal surface of the collagenous DS. White asterisks indicate a gap in the DS enabling communication to be made with AG. Note that not only the DS but also the AG interfere with picrosirius red polarization. **E** Another sample. Anti-CD31-immunostained specimen showing AG around Meckel’s cave. Horizontal section. General view. **F** Magnification of **E**. There is a large AG cluster adjacent to the posterior aspect of the cave and neighboring the dorsolateral wall (LW) of the cave. The vascular endothelium (dark brown) is revealed using an anti-CD31 reaction. AG are surrounded by venous channels (VC). Some non-specific DAB deposition is seen in the LW as an artifact. V, trigeminal nerve fascicles; V1, fascicles forming the ophthalmic nerve. Black arrowheads indicate remnants of the arachnoid mater. In **G**, another specimen shows a longitudinal AG cluster between the dorsolateral wall (LW) and the dural sleeve of Meckel’s cave. H&E stain. The AG cluster is extended anteriorly and joins the lateral wall of the parasellar space (cavernous sinus). The TG and V1 fascicles are located medially to the AG. Note the distance between the LW and AG. The LW extends anteriorly as the lateral wall of the lateral sellar compartment (LW-LSC). **H** Anti-CD31-immunostained specimen obtained from the sample shown in **G**. AG are surrounded by venous channels (VC). In this case, the space between the LW and AG is not an artifact but a vein (part of the laterotrigeminal venous system) lined with vascular endothelium (marked brown). Directions: A, anterior; P, posterior; L, lateral; M, medial. The scale bar shows 1 mm

The macroscopic observations were complemented with histological examination, which facilitated understanding of the relationships between the arachnoid granulations and their surroundings. When slices in the sagittal plane were assessed on one sample, a large cluster of granulations was found during cutting through the dorsolateral (superolateral) wall of MC, just under this wall (Fig. 4). Granulations were also seen under the dorsolateral wall of MC in all of the four specimens sectioned in the horizontal plane; first at the level of the anterior edge of the trigeminal ganglion (Fig. 5c, d), in the next two posteriorly to the ganglion (as an example, see Fig. 5e, f), and in another specimen between the dorsolateral wall and the dural sleeve of Meckel’s cave (Fig. 5g, h). In summary, clusters of arachnoid granulations were identified under the dorsolateral wall of MC in five of the six samples examined histologically.

**Fig. 6:**
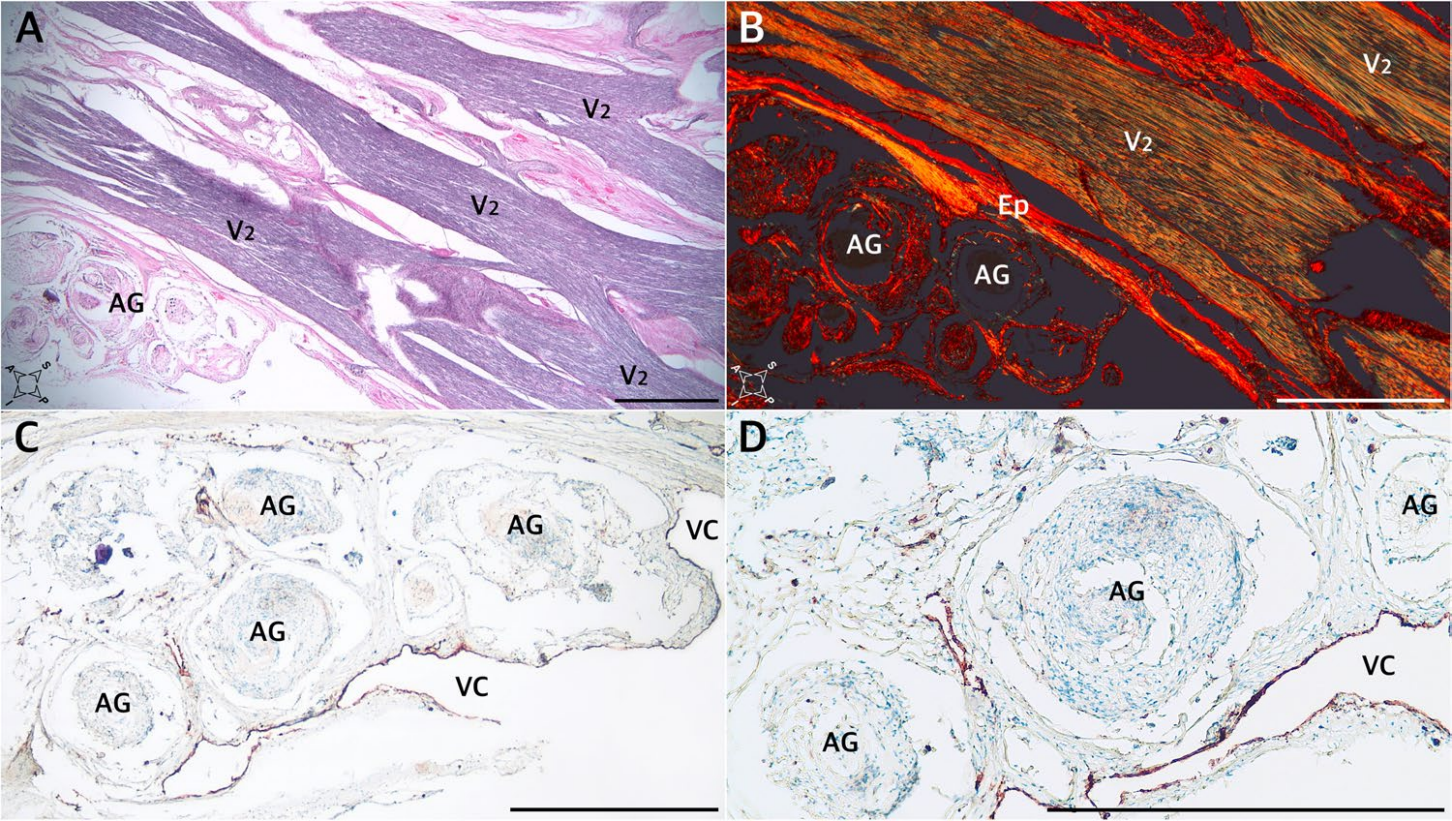
Arachnoid granulations (AG) adjacent to the maxillary nerve. Sagittal sections. **A** H&E stain. The granulations adhere to the maxillary nerve’s epineurium. The nerve is formed by numerous separate fascicles (V2). **B** The same specimen was stained with picrosirius red and seen under polarized light. In this specimen, AG are situated externally to epineurium (Ep) of the maxillary nerve. **C** shows anti-CD31 immunostaining of the same sample. **D** is a magnification of **C**. The presence of vascular endothelium is confirmed around the AG (dark brown reaction). AG are counterstained bluish by hematoxylin. VC, venous channel. Directions: A, anterior; P, posterior; S, superior; I, inferior. The scale bar shows 1 mm

**Fig. 7:**
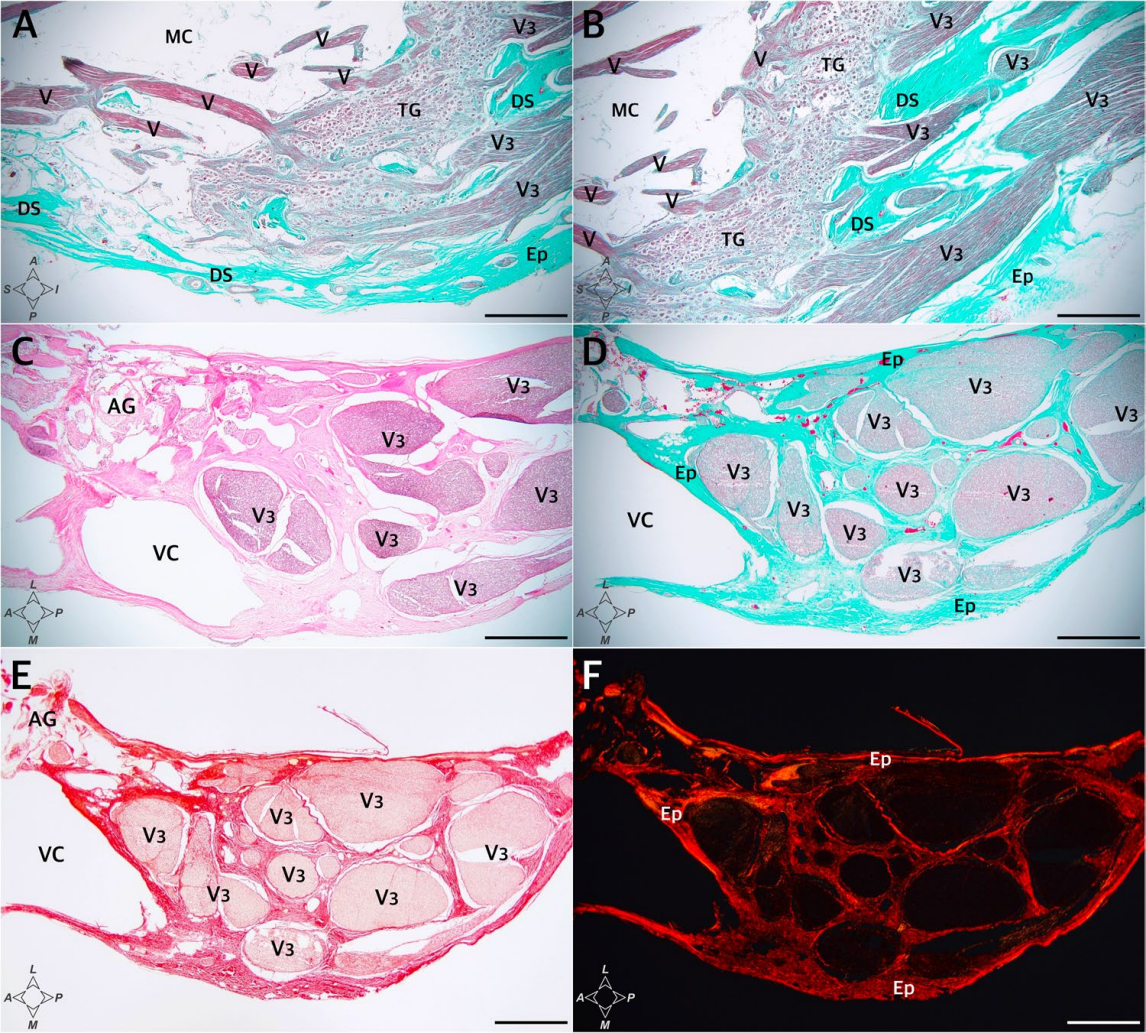
The anterior (rostral) end of MC at the level of the mandibular nerve’s origin. **A** and **B** show sagittal sections of the cave. The Masson–Goldner trichrome stain. There is a close relationship between the trigeminal ganglion and the dural sleeve (DS) of MC. The sleeve is not opened but constitutes a continuous entity with the cribriform area pierced by individual fascicles of the mandibular nerve (V3). The connective tissue that forms the DS is continuous with the nerve’s epineurium (Ep) and perineuria surrounding individual fascicles. **C–F** show cross-sections perpendicular to the long axis of the mandibular nerve (V3) obtained before the nerve reaches the foramen ovale. The internal architecture of the nerve is seen. **C** H&E stain. Arachnoid granulations (AG) are seen close to a sizeable venous channel (VC) adjacent to the nerve. **D** The Masson–Goldner trichrome stain. The mandibular nerve is surrounded by epineurium (Ep) composed of dense irregular connective tissue (stained light green). Numerous individual fascicles (V3) surrounded by connective tissue (light green) are visible in this section. **E** and **F** show the same specimen-stained picrosirius red and evaluated under normal and polarized light, respectively. Directions: A, anterior; P, posterior; L, lateral; M, medial; S, superior; I, inferior. The scale bar shows 1 mm

The detailed distribution patterns of arachnoid granulations and the areas where they were located varied greatly among individual histological slices. Arachnoid granulations near division V1 (ophthalmic) of the trigeminal nerve were found between the origins of separate nerve fascicles in one histological specimen sectioned horizontally (Fig. 5a, b), over the V1 division in one specimen sectioned sagitally, and at the level of the V1 origin (between the dural sleeve and dorsolateral wall of MC) in another specimen sectioned horizontally (Fig. 5g, h). There were also arachnoid granulations close to the maxillary (Fig. 6) and mandibular (Fig. 7) divisions in selected samples. As mentioned earlier, in two specimens sectioned horizontally, large clusters of arachnoid granulations extended to the posterior edge of Meckel’s cave (as an example, see Fig. 5e, f). In one sectioned sagitally, the granulations were captured around the portio minor of the trigeminal nerve. In all cases examined histologically, anti-CD31 immunostaining revealed vascular epithelium covering the clusters of arachnoid granulations (Figs. 3, 4, 5, 6), confirming that arachnoid granulations were surrounded by venous channels or lacunae. Arachnoid granulations were also stained positively with anti-podoplanin (D2-40) antibody (Fig. 4f).

### Observations on the meningeal architecture of Meckel’s cave

**Fig. 8:**
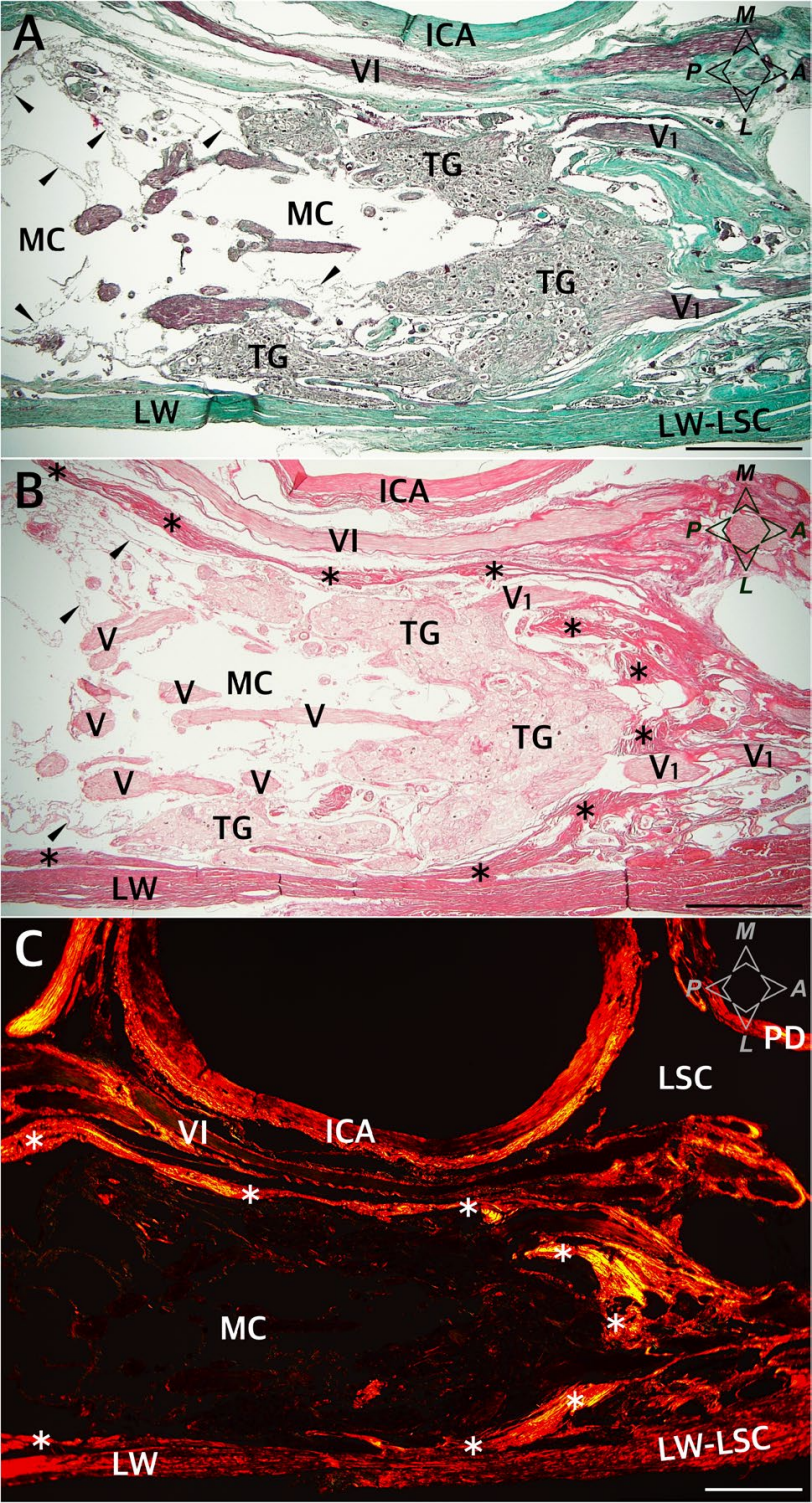
Topographical anatomy of Meckel’s cave (MC) and its meningeal relationships. Horizontal section of the right MC and posterior part of the lateral sellar compartment. **A** The Masson–Goldner stain. Connective tissue is stained light green. MC contains trigeminal nerve fascicles (V) surrounded by the arachnoid membrane (marked by black arrowheads). The dorsolateral wall (LW) of MC extends anteriorly as the lateral wall of the lateral sellar compartment (LW-LSC). **B** Picrosirius red stain. Specimen was photographed under normal light. The entire interior of MC is surrounded by a dural sleeve (marked by dark asterisks). The sleeve separates MC from the lateral sellar compartment, including the abducens nerve (VI) and parasellar segment of the internal carotid artery (ICA). At the anterior (rostral) end, the sleeve is pierced by individual fascicles of the ophthalmic nerve (V1). **C** Picrosirius red stain. The collagenous surroundings of MC are highlighted red and orange under polarized light. The DS, which interferes strongly with picrosirius red polarization, is marked by white asterisks in this photograph. PD, periosteal dura. Directions: A, anterior; P, posterior; L, lateral; M, medial. The scale bar shows 1 mm

The porus trigeminus is the dural entry point of the trigeminal nerve to Meckel’s cave (Fig. 3). It is bounded by the posterior petroclinoid dural fold superiorly, with the superior petrosal sinus running along this fold, over the porus (Fig. 3a, b, d, f). In two of the specimens (2/26; 7.7%), ossifications of the posterior petroclinoid dural fold were identified over the porus trigeminus. Histological examination of those specimens (after decalcification in 10% formic acid) revealed the heterotopic bony tissue within the fold (Fig. 3g, h). The vascular epithelium within the superior petrosal sinus was visualized using the anti-CD31 immunostain (Fig. 3f). An arachnoid mater surrounded the trigeminal nerve in the porus (Fig. 3c, d, f). MC contained fascicles of the trigeminal nerve, forming the trigeminal triangular plexus (Fig. 2f) surrounded by the arachnoid mater (Fig. 8a, b). There was also arachnoid mater between individual fascicles of the trigeminal nerve (Figs. 5c and 8a, b).

The outer part of the dorsolateral wall of MC was integral to the dura propria (meningeal layer of dura) covering the middle cranial fossa. It was continuous anteriorly with the outer part of the lateral wall of the cavernous sinus (Figs. 5g and 8). In all cases examined, there were tiny pores or trabeculations of highly variable location on the dorsolateral wall of MC (Fig. 1a). In histological specimens, veins were occasionally found along the lateral margin of MC, contributing to the laterotrigeminal venous system (Figs. 4e and 5e, f, h). Stratigraphic assessment of histological specimens sectioned sagitally revealed the dorsolateral wall of MC as a thick layer formed by interweaving bundles of collagen fibers arranged in various directions (Fig. 4a, b). The entire interior of MC was surrounded by a dural sleeve, which corresponds to the invaginated dura propria (meningeal layer of dura) of the posterior cranial fossa (Figs. 2; 7a, b; 8; and 9). The sleeve separated MC from the lateral sellar compartment (Fig. 8) and neighbored the petrolingual ligament, abducens nerve, and posterior vertical part of the parasellar internal carotid artery. At the anterior (rostral) end, the sleeve was pierced by individual fascicles of the trigeminal nerve’s primary divisions (Figs. 5a–d; 7a, b; 8; and 9). Arachnoid membrane was clearly observed on the inner side of the sleeve up to the trigeminal ganglion level (Figs. 5e, f and 8a, b). However, the arachnoid extended between the trigeminal nerve bundles to the trigeminal ganglion internal surface (Figs. 5c and 8a). Remnants of the arachnoid were also occasionally observed outside the trigeminal ganglion’s anterior pole (Fig. 5a–d).

**Fig. 9:**
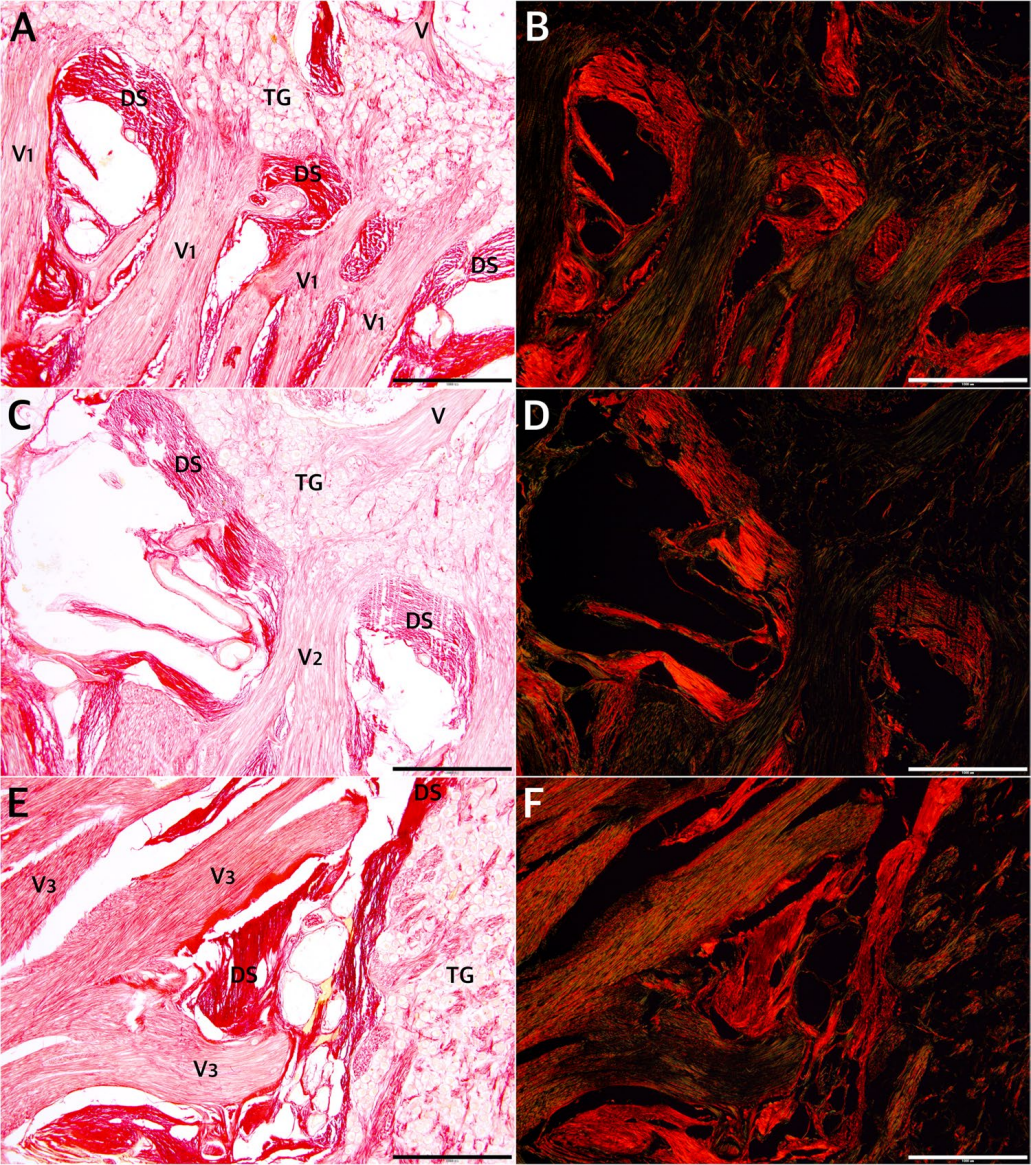
The rostral end of MC at the level of the origins of the ophthalmic (**A**, **B**), maxillary (**C**, **D**), and mandibular (**E**, **F**) nerves from the trigeminal ganglion (TG). Picrosirius red stain. Individual fascicles of the three main divisions pierce the dural sleeve (DS) of MC separately. **A, C, E** Photographs under normal light. **B, D, F** Specimen seen under polarized light. The collagenous network forming the DS is continuous with the perineuria surrounding the fascicles of the individual nerves. The collagenous structure of the DS strongly interferes with picrosirius red polarization and is highlighted in iridescent red in polarized light, as is the thinner perineurium surrounding individual fascicles. The intrafascicular connective tissue (endoneurium) appears green. The scale bar shows 1 mm

The connective tissue forming the dural sleeve of MC was fused with the cave’s dorsolateral wall of MC (see Fig. 8). The sleeve closely adhered to the trigeminal ganglion on the distal (anterior, rostral) end of MC (Figs. 5c, d; 7a, b; 8; and 9). This relationship was particularly clear in specimens stained with picrosirius red. Assessment of the origin of the trigeminal ganglion divisions in polarized light on picrosirius red-stained specimens revealed that individual nerve fascicles forming the primary (ophthalmic, mandibular, and maxillary) divisions pierced the dural sleeve separately (Fig. 9). These histological observations therefore indicate that the dural sleeve (dural envelope) of MC was not open where the trigeminal divisions emerged but constituted a continuous entity with areas perforated by relatively small pores containing individual nerve fascicles derived from the trigeminal ganglion. The connective tissue forming the sleeve was not only continuous with the epineurium covering all the primary divisions but also shifted to the perineuria surrounding individual fascicles of the three main trigeminal nerve divisions (Figs. 7 and 9). After the primary divisions left MC inside, the periosteal dura soon joined the outer shell for the mandibular and maxillary nerves and fused with the epineuria of those nerves (as an example, see Fig. 7c–f). The motor part of the trigeminal nerve (portio minor) crossed the inferomedial surface of the ganglion and then joined the mandibular division (Fig. 2). Although not “opened” on the rostral end, the dural sleeve of Meckel’s cave contained pores communicating with arachnoid granulations (as shown in Fig. 5c, d).

**Fig. 10:**
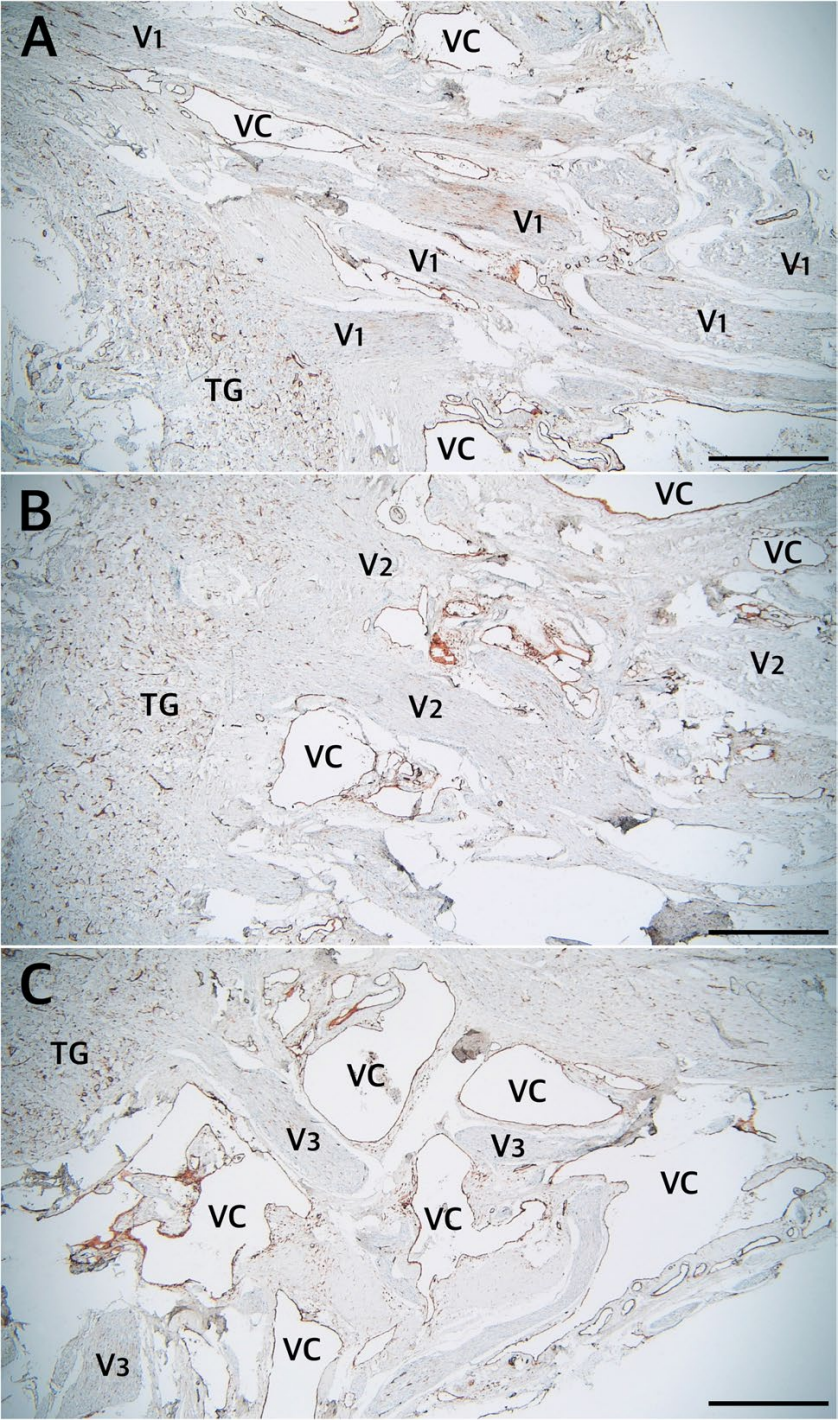
The rostral end of MC at the levels of origin of the ophthalmic (**A**), maxillary (**B**), and mandibular (**C**) nerves from the trigeminal ganglion (TG). Anti-CD31 immunostaining showing the vascular epithelium. Individual fascicles of the three main divisions are surrounded by numerous venous channels (VC) in their initial parts. The venous channels are located outside Meckel’s cave. The scale bar shows 1 mm

There were numerous CD31-positive reactions within the trigeminal ganglion, suggesting a dense network of tiny vascular vessels. On the external surface of the dural sleeve of MC, numerous venous channels or lacunae lined with epithelium (marked with anti-CD31 immunostaining) were revealed between the origins of the separate nerve fascicles forming primary divisions of the trigeminal nerve (Figs. 5b and 10). The arachnoid granulations were located externally to the dural sleeve, as observed in picrosirius red-stained specimens (Fig. 5c, d).

## Discussion

### Arachnoid granulations and venous spaces

In the entire literature on MC, only few papers have attempted to describe adjacent arachnoid granulations topologically. A detailed review by Bond et al. [3] summarized current knowledge. Those authors referenced only four papers that mention the arachnoid granulations around MC and the trigeminal nerve’s main divisions. The cited authors describe the arachnoid granulations only in the superior wall of MC or only around the medial wall. However, the histological evidence presented in this paper revealed granulations on each side of MC. Kawase et al. [19] mention arachnoid villi protruding into the venous drainage route along the mandibular nerve, located on the medial wall of MC. Kehrli et al. [20] describe arachnoid granulations in humans as “easily observable along arachnoid and dural sheath of the cranial nerves running in the parasellar lodge,” but they did not find arachnoid granulations or villi in the olive baboon *Papio anubis*. Kehrli et al. [20] described them in relation to the trochlear and oculomotor nerves. These authors also provide more details of granulations that “often invaded the parasellar lodge, between the V1 and V2, or sometimes, seeped into the peripheral sheath of these nerves and could be observed among their fascicles.” Janjua et al., in turn, mentioned that arachnoid granulations can be found predominantly lateral to V3 within the venous pool belonging to the so-called laterotrigeminal venous system. These findings were specified in both gross and microanatomical examinations of our samples; the granulations were most often located between divisions V2 and V3 of the trigeminal nerve (69.2% of specimens) and less frequently between V1 and V2 (42.3%).

From a clinical point of view, the vicinity between arachnoid granulations and petrous temporal bone can be relevant. Our study revealed arachnoid granulations posterior to trigeminal ganglion and V3 division in 30.8% of cases. These clusters were particularly close to the bony surface of the petrous pyramid. The proximity between arachnoid granulations and the trigeminal impression provides a potential anatomical background for the petrous temporal bone erosion in hypertrophy of these granulations. According to Yew et al. [40], “Erosion of the temporal bone by AGs is not a rare occurrence in the population and becomes increasingly prevalent with age.”

Chen et al. [5] studied arachnoid granulations of the middle cranial fossa and reported the foramen rotundum, foramen ovale, and cavernous sinus (along the inside of Meckel’s cavity) as their potential locations. The cited authors made histological observations consistent with the data in our study, concluding that “In serial sections, the protuberances were seen to occupy some definite apertures in the dural lining, always lying close either to a vascular channel or to the venous plexus.” Our study also confirms direct relationships between arachnoid granulations and the venous system around the trigeminal ganglion. The venous system around the petrous temporal bone (including MC and trigeminal impression) is complex; transpetrosal veins and venous channels are linked to the superior petrosal sinus [8, 34]. Williams et al. [35] examined magnetic resonance imaging of the trigeminal ganglion and nerve and the perineural vascular plexus, correlating with cadaver specimens. They describe the venous relationships around MC as “a constant and extensive pericavernous venous plexus” located in a dural envelope, in all cases surrounding the trigeminal ganglion. Venous channels were observed along the lateral margin of the sella, the medial aspect of the middle fossa, the inferior orbital fissure, and the foramina ovale, rotundum, and spinosum [35]. Simões [34] systematized the venous vessels located along the lateral margin of the trigeminal cave and described their arrangement as the “laterotrigeminal venous system.” In the present study, remnants of this system were revealed around the dorsolateral wall of MC by histological examination. Arachnoid granulations occasionally accompanied the venous channels of this system (see Figs. 4e and 5e, f, h). Kehrli et al. [21] stressed that the foramen ovale is surrounded by a very rich venous supply and the mandibular nerve. They often found small dural venous channels between the maxillary and ophthalmic nerves; the space between the two nerves sometimes contained arachnoid granulations. Our study also confirmed the presence of vascular epithelium from numerous venous channels among individual fascicles forming the divisions of the trigeminal nerve. These channels were densest around the V3 division. The aforementioned venous channels can also be considered part of an extradural neural axis compartment [30].

Whether the numbers and sizes of the main clusters of arachnoid villi around MC are age-related is not clear. Kehrli et al. [20] only mention that arachnoid granulations are found, although less abundantly, in young human adults. Chitterman et al. [7] hypothesize that arachnoid villi can potentially develop throughout one’s adult life. This assumption is consistent with the observations of Yew et al. [40], who found an increasing prevalence of arachnoid granulations penetrating the temporal bone with advancing age, achieving 28% for patients 50 years and older. Our sample was taken from body donors between 47 and 84 years old.

Summarizing the information from the present study, clusters of arachnoid granulations of different sizes occupied variable positions along the trigeminal ganglion around the origins of the ophthalmic, mandibular, and maxillary divisions of the trigeminal nerve. This study also paid attention to the relation of arachnoid granulations to selected surgical triangles, which has not been discussed in the literature before [14, 22, 37]. Histological examination revealed arachnoid granulations on both the lateral and medial sides of MC and posterior and anterior to the cave. Granulations could make an outflow of cerebrospinal fluid possible from inside Meckel’s cave to the venous channels surrounding it [3, 19]. This role could suggest that the presence of arachnoid granulations around MC is a normal physiological condition. In recent years, there have been detailed and insightful reports on cerebrospinal fluid circulation in MC and the lateral sellar compartment. The possible role of the cavernous sinus veins in cerebrospinal fluid absorption was examined by Johnston et al. [17]. They infused a colored silastic material (Microfil) into a sheep’s subarachnoid space (cerebellomedullary cistern) and then examined the relevant tissue macroscopically and microscopically. They found that cerebrospinal fluid from the cerebellomedullary cistern could gain access to absorption sites by various pathways. The cited authors found Microfil in the spaces surrounding the cavernous sinus venous network, in the adventitia of the internal carotid artery, adjacent to the pituitary gland, within the endoneurial spaces of the trigeminal nerve, and in lymphatic vessels emerging from the epineurium of that nerve.

### Meningeal architecture of Meckel’s cave

Bond et al. [3] indicate that the complex arrangement of delicate meningeal structures surrounding MC is extremely difficult to study using gross anatomical techniques. Observations included in the present paper supplement existing models of the meningeal architecture of MC. Complex relationships between the dural sleeve of MC and the trigeminal ganglion with its branches were revealed and explained using histological methods, which allowed the microanatomy to be traced in detail.

The dural covering surrounding MC is formed by the invaginated dura propria of the posterior cranial fossa. It is closely adherent to the dura propria of the middle cranial fossa (externally, at the side of MC dorsolateral wall) and to the periosteal dura of the trigeminal impression (at the internal site) [18, 26]. Janjua et al. [16] provided elegant and detailed descriptions of the dural relationships around the parasellar compartment and MC. They stress that “At the foramen ovale, the periosteal and meningeal dura separated, with the former passing under the trigeminal nerve complex forming the floor of the parasellar region while the latter overlaid the entire parasellar region, including MC.” A thin, delicate arachnoid pocket extends from the prepontine cistern. In our series, the arachnoid membrane ranged from the porus trigeminus to the trigeminal ganglion. There were also traces of arachnoid trabeculations between fascicles forming the trigeminal triangular plexus. We observed arachnoid mater up to the level of the trigeminal ganglion internal surface. However, remnants of the arachnoid were also incidentally detected outside the trigeminal ganglion’s anterior pole (see Fig. 5c).

Our understanding of the relationships of the meninges to the trigeminal ganglion and its divisions changes as new research data are obtained. Three antithetical “open-ended three-fingered glove” models of the relationships of the dura and arachnoid mater to MC were discussed by Kehrli et al. [21] and Bond et al. [3]. In the first model, all dura and arachnoid layers loosely surround the trigeminal ganglion and its three divisions until they pass through the skull base. The second has the same dural relationships, but the arachnoid mater stops at the level of the trigeminal ganglion. According to this concept, the dura loosely surrounds the trigeminal ganglion divisions. In the third model, MC is surrounded by the separate dural sleeve (invaginated dura propria of the posterior cranial fossa) and arachnoid mater. Both coverings fuse with the rostral end of the trigeminal ganglion and the dura is continuous with the epineuria of the three main trigeminal nerve divisions. Detailed illustrations of all three models are presented by Kehrli et al. [21] and Bond et al. [3]. In the original version, the dural sac of MC was considered open-ended, which was incorrect. The model was modified by Li et al. [26], who explored the dural sheath of the trigeminal nerve using microsurgical dissection and modified the “three-fingered glove model” of the trigeminal cave, introducing the concept of a “cribriform area” as the site of origin of the trigeminal ganglion’s primary divisions. This was corroborated by our results.

It should also be mentioned here that the arrangement and structure of the walls of MC are still controversial. Various models have been proposed, with the dural wall of MC composed of either a single dural layer or a more complex arrangement involving the dura propria (meningeal layer of dura) of the posterior cranial fossa invaginated internally between the meningeal and periosteal dura of the middle cranial fossa [3]. The most recent model has been well corroborated and is also supported by the present findings. Developmental concepts concerning the meningeal architecture of MC have been extensively discussed in the literature and are therefore omitted from this discussion. Kehrli et al. [21] showed, following an embryological investigation, that “The posterior fossa dura propria makes a pouch which forms the cavum trigeminale” and “There is no contact between these nerves and arachnoid except the arachnoid granulations, which can accompany the nerves or even penetrate their peripheral sheath.” Contrary to our findings, those authors stated that the dural sleeve (pouch) of MC “clearly ends at the posterior part of the trigeminal ganglion and we have never been able to observe dura and arachnoid around its branches” [21]. The last statement seems confusing in view of our recent histological observations of adult cadavers. The cited authors probably wanted to emphasize the clear border of the dural sleeve and the fact that it is distinct from the nerve’s perineurium. However, histologically, when the distribution of connective tissue is traced on picrosirius red- or trichrome-stained specimens, there is no clear borderline between the connective tissue forming the dural sleeve (dural pouch) of MC and the perineuria of the trigeminal nerve’s primary divisions. Observations similar to ours were presented by Li et al. [26], whose cadaveric microsurgical dissections revealed that “the dural sheath of the trigeminal nerve is meningeal dura in origin and composed of Meckel’s cave and the peripheral sheaths. The peripheral sheath is a direct continuation of Meckel’s cave, but separated from the latter by a cribriform area from where the nerve rootlets pass through.” A detailed histological examination of the rostral end of the dural sleeve of MC confirmed that connective tissue at this location stays in touch (mixes) with the perineuria of the three main trigeminal ganglion divisions, as demonstrated in our study using trichrome and picrosirius red stainings. Liang et al. [24, 25] confirmed this using another research approach—plastinated sections examined at both macroscopic and microscopic levels—for assessing the trigeminal nerve and parasellar compartment. Although this observation challenges classical descriptions of three separate openings for divisions V1–V3, it is reliable and clinically valid. The continuity between the dural sleeve and perineural connective tissue can be considered a potential route for the spread of perineural tumors in this region [26].

### Porus trigeminus

The porus trigeminus is a dural entry point for the trigeminal nerve, which forms a natural connection between the posterior and middle fossas [32]. Its shape is variable and it is located between the petrous apex and the posterior petroclinoid dural fold. The superior petrosal sinus travels over the porus trigeminus. The junction between the superior petrosal sinus and the lateral sellar venous complex is located near the superomedial aspect of the porus trigeminus, while the inferior petrosal sinus joins the basilar plexus at the inferomedial aspect of the porus [32].

There are occasional ossifications or calcifications near MC and the porus trigeminus, such as ossifications of the posterior petroclinoid dural fold. Such ossifications can extend and involve a variable area of the petroclival dura [36, 37]. Kimball et al. [22] describe such ossifications as an anatomical variation causing the trigeminal nerve to traverse a bony foramen as it enters Meckel’s cave. Those authors introduced the term “petroclinoid bone” for the osseous bridge over the trigeminal nerve’s dural entry point. According to Inal et al. [13], it can be challenging to differentiate between the calcifications of the posterior petroclinoid dural fold and the underlying petrosphenoidal ligament, which are more accessible in 3D views. The frequency of complete ossification of the posterior petroclinoid dural fold differs among authors. In our previous report on the inferomedial paraclival triangle, two of the 46 sides examined showed such ossification [37]. Kimball et al. [22] found complete ossifications of the posterior petroclinoiddural fold in three of 15 wet specimens. Radiologically, calcification at the petroclival region involving the posterior petroclinoid dural fold is described even more frequently. Inal et al. [13] reported 26.6% partial and 5.2% complete calcifications on the right side and 29.5% partial and 4.6% complete calcifications on the left, more frequently detected in older patients. Cederberg et al. [4] detected complete ossification of the petroclinoid folds radiographically in 9% of subjects, which is consistent with our recent sample (7.7%). It is also worth mentioning the os supra petrosum of Meckel, a small ossicle occasionally located on the anterosuperior surface of the petrous bone and just anterior and medial to the trigeminal ganglion [9]. Unexpected ossifications of the dura can alter neurosurgical access. To take a specific example, Seoane and Rhoton [33] stress that the suprameatal extension of the retrosigmoid approach can be extended by opening the superior petrosal sinus as it crosses the porus trigeminus’s upper margin. Ossifications over porus trigeminus or anatomical variations of the porus can hinder such maneuvers. For this reason, careful preoperative evaluation of the anatomical conditions around MC is needed [29, 33].

### Study limitation and further research perspectives

From a technical point of view, immunohistochemistry should be performed on the freshest material possible. For legal reasons, the histological material involved in this study could not be obtained within 24 h after the donor’s death. However, we decided to include immunohistochemistry because its evidential power is solid. Some minor artifacts such as non-specific background tissue staining by DAB do not affect the scientific value of the results since the epithelium was clearly marked.

Another limitation regarding the detailed evaluation of the arachnoid granulations is that electron microscopy could not be included among the scientific tools at this stage of the research. Thorough research on communications between the inside of Meckel’s cave and adjacent arachnoid granulations could be conducted. The immunohistochemical characteristics of arachnoid granulations should also be determined.

As the medical history of the body donors was unknown, the authors of this research have no information about possible neurological symptoms in the sample group. It also remains uncertain if a reduced of increased number of arachnoid granulations around MC can be linked to specific clinical symptoms, e.g., trigeminal neuralgia. The phenomenon of arachnoid villi overgrowth with age or increased intracranial pressure [7, 40] may also be significant in this context. Further studies of the cerebrospinal fluid circulation within MC should be conducted to highlight this issue. The study is purely descriptive. Therefore, it cannot be determined whether the number of arachnoid granulations around MC influences cerebrospinal fluid circulation within the cave.

Mezey et al. [28] presented, inter alia, lymphatic endothelial markers between the nerve fibers and fascicles of the cranial nerves. In recent years, because specific markers for lymphatic endothelial cells have been found, the lymphatics of the crucial skull base regions have been studied [38, 39]. Further research on the lymphatic vessels around Meckel’s cave is another exciting perspective. All the more so, as what Yağmurlu et al. suggested, “AGs may function as a component of the CNS lymphatic drainage, serving as a surrogate for lymph nodes in the CNS” [39].

There are also suggestions that meningiomas may originate from cells of arachnoid villi [7, 11, 12]. Thus, further research on arachnoid granulations around MC should include material harvested post-mortem from healthy body donors of various ages and those with pathological conditions around MC. However, although the present work has some limitations, it supplements previous knowledge and could inspire further research.

## Conclusions

The meningeal architecture around MC has a complex multilayer arrangement with a collagenous dural sleeve closely related to the trigeminal ganglion and its primary divisions. Trichrome and picrosirius red staining demonstrated that the open-ended three-fingered glove model is a simplification. The sleeve at the rostral end of Meckel’s cave is continuous with the cribriform area at the point of origin of the ophthalmic, maxillary, and mandibular nerves. Numerous individual fascicles forming the primary divisions pierce the dural sleeve separately. The connective tissue of the dural sleeve is continuous with both epineurium and perineurium within the divisions of the trigeminal ganglion. Arachnoid granulations are typically found around MC.

## Data Availability

Data are contained within the article.
